# Serum Lipocalin-2 Levels Are Increased and Independently Associated With Early-Stage Renal Damage and Carotid Atherosclerotic Plaque in Patients With T2DM

**DOI:** 10.3389/fendo.2022.855616

**Published:** 2022-04-25

**Authors:** Jing Gan, Yu Zheng, Qiongli Yu, Yingchao Zhang, Wei Xie, Yaru Shi, Ning Yu, Yu Yan, Zhuofeng Lin, Hong Yang

**Affiliations:** ^1^ The 2nd Affiliated Hospital of Wenzhou Medical University, Wenzhou, China; ^2^ School of Pharmaceutical College, Wenzhou Medical University, Wenzhou, China; ^3^ The First Affiliated Hospital of Wenzhou Medical University, Wenzhou, China; ^4^ Laboratory Animal Center of Wenzhou Medical University, Wenzhou, China; ^5^ The 3rd Affiliated Hospital of Wenzhou Medical University, Wenzhou, China

**Keywords:** lipocalin-2, carotid atherosclerotic plaque, early-stage renal damage, type 2 diabetes mellitus, cardiovascular disease

## Abstract

**Objectives:**

Diabetic nephropathy (DN), one of the major complications of diabetes mellitus, is the major cause of end-stage renal failure that finally increases the risk of cardiovascular disease and mortality. The aim of this study is to explore the relationship between serum lipocalin-2 (LCN-2) levels and DN and carotid atherosclerotic plaque (CAP) in patients with type 2 diabetes mellitus (T2DM).

**Methods:**

We have performed a prospective study of 749 T2DM patients with or without DN. Blood samples were collected and used to test serum LCN-2 levels, renal function, as well as biochemical parameters. CAP in these subjects was determined by ultrasonography.

**Results:**

In these 749 subjects with T2DM, an increased morbidity of CAP was observed in T2DM patients with DN as compared with those without this complication (*P* < 0.05). Interestingly, serum LCN-2 levels were significantly increased in T2DM patients with DN or CAP compared with T2DM alone [97.71 (71.49-130.13) *vs.* 77.29 (58.83-115.05) ng/ml, *P* < 0.001]. In addition, serum LCN-2 levels in T2DM patients with DN and CAP were significantly higher than that of T2DM patients with DN or CAP [131.37 (101.43-182.04) *vs.* 97.71(71.49-130.13) ng/ml, *P* < 0.001]. Furthermore, serum LCN-2 levels were positively correlated with hemoglobin A1c, systolic blood pressure, hypertension, CAP, and DN, as well as renal function factors including uric acid, creatinine, the estimated glomerular filtration rate, and urinary albumin-to-creatinine ratio, respectively (*P* < 0.05), but negatively correlated with HDL-c (*P* < 0.05). The multinomial logistic regression analysis showed that serum LCN-2 was independently associated with DN and CAP in patients with T2DM after the adjustment for risk factors (*P* < 0.001).

**Conclusions:**

Early-stage renal damage is a risk factor associated with the incidence of CAP in patients with T2DM. Serum LCN-2 is significantly increased and associated with early-stage renal damage and the incidence of CAP in patients with T2DM.

## Introduction

The mortality of cardiovascular disease (CVD) has dramatically increased and continued to rise worldwide in the recent decade (1). Early intervention could prevent progression and facilitate positive outcomes; however, it remains to be a huge public health challenge to address CVD. Diabetes has long been considered as an independent risk factor for CVD, easily causing varying degrees of atherosclerosis (AS) in blood vessels (2). In addition, diabetes mellitus was considered as the leading cause of chronic kidney disease and caused approximately 1.5 million deaths in 2016 (3). Moreover, previous clinical studies demonstrated that the cardiovascular mortality rate of patients with diabetic nephropathy (DN) was significantly higher than that of patients without DN (4, 5); thereby, DN may be related to macrovascular lesions and promote AS to a certain extent.

Recent studies demonstrated that lipocalin-2 (LCN-2) in serum and unstable atherosclerotic plaques in patients with AS is significantly higher than that in the normal population, and is positively correlated with the thickness of intima media in patients with AS, suggesting that LCN-2 may be involved in the formation of AS (6, 7). LCN-2, mainly released from granules of activated neutrophils and also known as neutrophil gelatinase-associated lipocalin (NGAL), is recognized as an acute-phase protein induced by multiple stresses (8). Previous studies showed that LCN-2 levels are markedly increased in patients with CVD, including AS and heart failure, but not in healthy subjects (9). Furthermore, LCN-2 is found to be expressed in macrophages, smooth muscle cells, and endothelial cells in human carotid plaques (10, 11). Therefore, NGAL is considered as a potential circulating biomarker of plaque vulnerability in patients with carotid atherosclerosis (CAS) (12). On the other hand, some studies showed the excellent performance of urinary or plasma LCN-2 levels as a prognostic biomarker of acute kidney injury or acute heart failure (13, 14). However, whether LCN-2 is related to the development of carotid atherosclerotic plaque (CAP) in T2DM patients with DN remains unclear.

In present study, we explored the relationship between LCN-2 and DN and CAP in patients with T2DM. Our data demonstrated that early-stage renal damage is a risk factor associated with the incidence of CAP in patients with T2DM, and serum LCN-2 levels are significantly associated with early-stage renal damage and the incidence of CAP in patients with T2DM.

## Materials and Methods

### Patient’s Selection

A total of 749 patients with T2DM were enrolled in this project in the Department of Endocrinology, Ruian People’s Hospital from July 1, 2019 to June 30, 2021. The clinical diagnosis of T2DM referred to the diagnosis and classification standards of diabetes proposed by the World Health Organization (WHO) in 1999: diabetes symptoms, plasma glucose level at any time of 11.1 mmol/L (200 mg/dl), fasting blood glucose (FPG) levels of 7.0 mmol/L (126 mg/dl), or 2-h plasma glucose levels of 11.1 mmol/L (200 mg/dl) in an oral glucose tolerance test (15). In this study, DN was defined as the patients with “early-stage renal damage” on the basis of Kidney Disease Outcomes Quality Initiative (KDOQI) Clinical Practice Guidelines. All the T2DM patients with the early-stage renal damage (DN) were diagnosed according to the urinary albumin-to-creatinine ratio (UACR) (if ≥30 mg/g) calculated from first-spot morning urine collections based on KDOQI Clinical Practice Guidelines (16). All of the subjects enrolled in the study were diagnosed with T2DM with a history of diabetes diagnosis or currently used oral hypoglycemics or insulin. Subjects with diabetic ketoacidosis, other cardiovascular and cerebrovascular diseases, abnormal liver function, macroalbuminuria or decreased renal function, other types of nephropathy, acute renal injury, uremic dialysis, hyperthyroidism, adrenocortical hyperfunction, pregnancy, and acute infection in the near future were excluded. This study was approved by the Human Ethics Committee of the Ruian People’s Hospital. All participants agreed and signed the informed consent forms.

### Clinical Data Collection

Clinical and biochemical data were obtained. Briefly, baseline information on demographics, health-related habits, the medical history, and current use of medicines were collected using a standardized questionnaire. Smoking or drinking was classified as “current” (smoking or drinking in the past 6 months or quit smoking or drinking within the past 6 months), “former” (cessation of smoking or drinking for more than 6 months), or “never”. Exercise habits were grouped according to frequency per week (less than or equal to 2 times/week or more than or equal to 3 times/week, with the latter indicating that the individual has physical activity). Anthropometric parameters were measured by trained staff. Body height and weight were recorded to the nearest 0.1 cm and 0.1 kg, respectively, while participants were wearing light indoor clothing without shoes. The body mass index (BMI) was calculated as kg/m^2^. Blood pressure, including systolic blood pressure (SBP) and diastolic blood pressure (DBP), was measured three times, which were taken at 5-min intervals, and a mean value was calculated; however, if the difference between any two readings was greater than 10 mmHg, the two closest measurements were used.

### Blood Sample Collection

Following an 8–12 h period of fasting, the whole blood samples obtained from all participants were used to isolate serum samples and then stored in -80°C for further analysis.

### Measurement of Lipocalin-2 and Biochemistric Parameters

Serum LCN-2 concentrations were determined using enzyme-linked immunosorbent assays (Antibody and Immunoassay Service, HKU, Hong Kong). Urinary creatinine was detected by the sarcosine oxidase-peroxidase antiperoxidase (PAP) method (7600-120 Hitachi automatic biochemical analyzer; Hitachi, Tokyo, Japan). Immunonephelometry was used to detect urinary albumin (BN II System; Siemens, Erlangen, Germany). UACR was calculated by dividing the urinary albumin by the urinary creatinine levels. The calculation formula of UACR (mg/g) = urinary microalbumin (mg/L) ÷ urinary creatinine (μmol/L) × 8,840. Biochemistric parameters including fasting plasma glucose (FPG), triglyceride (TG), total cholesterol (TC), high-density lipoprotein cholesterol (HDL-c), low-density lipoprotein cholesterol (LDL-c), serum uric acid (Sua), blood urea nitrogen (Bun), and serum creatinine (Scr) levels were determined using an auto biochemical analyzer (Cobas c702; Roche, Shanghai, China). Hemoglobin A1c (HbA1c) was measured with a high-performance liquid graphy method (BioRad, Hercules, CA, United States). The CKD Epidemiology Collaboration formula was used to calculate the estimated glomerular filtration rate (eGFR) (17).

### Assessment of Carotid Atherosclerotic Plaque

Ultrasound B-mode imaging (Philips HDI 5000 ultrasound system equipped with a 7.5 MHz probe) was annually performed to evaluate CAP. Intima-media thickness was measured at the point of approximately 1.5 cm away from the distal part of the bifurcation of the common carotid artery. CAP is defined as a focal region with a thickness of >1.5 mm as measured from the media-adventitia interface to the lumen-intima interface or as the presence of focal wall thickening that is at least 50% greater than that of the surrounding vessel wall (18). At the beginning of the study, the plaque score was measured and recorded. The plaque score was measured according to the Grouse criteria (19).

### Statistical Analysis

Data are reported as median (interquartile range), or mean ± standard deviation were given to describe continuous variables; absolute numbers and percentages were used to describe categorical variables. The continuous data among patients were compared with one-way ANOVA (the variables analyzed conform to the normality or homogeneity of variance test) or the nonparametric Kruskal–Wallis test (the variables analyzed do not conform to the normality or homogeneity of the variance test), and categorical data were analyzed by the χ^2^ test. Multiple comparisons were analyzed by one-way ANOVA followed by Tukey’s Honestly Significant Difference (HSD) test, the Kruskal–Wallis test, and Dunn’s test.

An age-adjusted partial correlation analysis was performed to determine the association of renal function indexes with DN or CAP. Moreover, an age- and T2DM duration-adjusted partial correlation analysis was performed to determine the association of LCN-2 with potential factors. The correlation coefficient for confounding factors was calculated. Binary logistic regression analysis was applied to determine the association between early-stage renal damage and CAP. Additionally, a multinomial logistic regression model was used to examine the association between LCN-2 and DN and CAP. Potential confounders were adjusted in different models. Statistical analyses were performed using SPSS 26.0 (IBM) software. Two-sided *P*-values <0.05 were considered as indicating statistical significance.

## Results

### Diabetic Nephropathy Increases the Incidence of Carotid Atherosclerotic Plaque in Patients With Type 2 Diabetes Mellitus

A total of 749 T2DM patients were enrolled in this study: 381 subjects with DN (50.87%), 316 subjects with CAP (42.19%), and 184 subjects with both DN and CAP (24.57%). Moreover, the prevalence of DN in 749 patients with T2DM was 50.87%, and the prevalence of CAP in T2DM subjects with DN is significantly higher than those without DN (48.29% vs. 35.87%, *P* = 0.001, **Table 1**), suggesting that DN may be a risk to promote the morbidity of CAP in patients with T2DM.

**Table 1:** Relationship between DN and CAP.

	Non-CAP	CAP	*χ* ^2^	*P*
Non-DN	236 (64.1%)	132 (35.9%)	11.848	0.001
DN	197 (51.7%)	184 (48.3%)		

### Serum Lipocalin-2 Levels Are Significantly Increased in Type 2 Diabetes Mellitus Patients With Diabetic Nephropathy and/or Carotid Atherosclerotic Plaque

To investigate whether LCN-2 is related to the pathogenesis of CAP in patients with T2DM, we first explored the influence factors of CAP in these T2DM subjects. As shown in **Table 2**, an increased manner of age was observed in these T2DM subjects with DN and/or CAP compared with those without these complications. Consistent with the change of age, a higher prevalence of hypertension was observed in these individuals, following a longer duration of T2DM and a higher smoking and drinking rate. However, no obvious differences of gender and BMI, as well as blood lipid parameters including TC, TC, HDL-c, and LDL-C, were observed in these T2DM subjects with or without these DN or CAP complications (all *P* > 0.05).

**Table 2:** Comparison of clinical characteristic in T2DM patients with or without relevant compliations including DN and CAP.

Variables	Total (n = 749)	T2DM group (n = 236)	DN or CAP group (n = 329)	DN with CAP group (n = 184)	P
LCN-2, ng/ml	99.59 (70.67-137.28)	77.29 (58.83-115.05)	97.71 (71.49-130.13)	131.37 (101.43-182.04)	**<0.001**
UACR, mg/g	19.08 (9.62-58.71)	11.35 (6.98-17.76)	19.08 (9.62-52.08)	68.90 (32.21-277.38)	**<0.001**
Age, years	54.0 (47.0-60.0)	49.5 (41.0-56.0)	54.0 (47.0-60.0)	58.0 (54.0-63.0)	**<0.001**
Male, %	73	71.7	72.0	76.6	0.444
Current smoking, %	45.2	33.9	45.1	59.8	**<0.001**
Current drinking, %	32.8	25.0	31.3	45.7	**<0.001**
Physical activity, %	11.5	9.7	11.6	13.6	0.472
Hypertension, %	44.7	30.5	43.5	65.2	**<0.001**
Antihypertensive therapy, %	7.8	14.0	21.3	51.6	**<0.001**
ACEI/ARB, %	47.3	31.8	47.1	67.4	**<0.001**
T2DM duration, months	11.71 (7.09-122.00)	7.59 (6.44-9.39)	20.0 (6.84-107.50)	130.0 (85.50-207.75)	**<0.001**
BMI, kg/m^2^	25.40 (23.3-27.4)	25.40 (23.30-27.55)	25.3 (23.3-27.6)	25.35 (23.3-27.1)	0.903
FPG, mmol/L	7.53 (6.22-9.46)	7.65 (6.45-9.40)	7.50 (6.19-9.43)	7.40 (6.03-9.85)	0.918
HbA1c, %	8.38 (7.25-10.42)	8.44 (7.23-11.08)	8.26 (7.21-10.27)	8.57 (7.33-10.07)	0.593
SBP, mmHg	134.18 ± 19.92	128.85 ± 17.40	133.34 ± 18.37	142.52 ± 22.82	**<0.001**
DBP, mmHg	76.28 ± 11.17	75.81 ± 10.06	76.77 ± 11.38	76.04 ± 12.15	0.568
TG, mmol/L	1.57 (1.08-2.26)	1.50 (1.07-2.20)	1.61 (1.13-2.32)	1.53 (1.03-2.31)	0.817
TC, mmol/L	4.60 (3.90-5.35)	4.56 (3.89-5.41)	4.63 (3.98-5.30)	4.67 (3.84-5.40)	0.962
HDL-c, mmol/L	1.03 (0.88-1.22)	1.03 (0.88-1.23)	1.02 (0.88-1.22)	1.03 (0.86-1.20)	0.966
LDL-c, mmol/L	2.82 (2.14-3.41)	2.81 (2.22-3.35)	2.85 (2.17-3.40)	2.73 (2.00-3.47)	0.607
Sua, μmol/L	326.0 (272.0-392.0)	313.0 (266.3-387.0)	331.5 (274.0-394.8)	332.0 (271.25-394.50)	0.284
Bun, mmol/L	5.24 (4.40-6.27)	5.08 (4.29-5.95)	5.17 (4.38-6.24)	5.6 (4.73-6.78)	**<0.001**
Scr, mg/dl	69.0 (62.0-75.75)	69.0 (62.0-74.75)	68.5 (62.0-75.0)	70.5 (64.0-80.75)	0.032
eGFR, ml/min/1.73 m^2^	105.63 (97.42-113.36)	109.14 (100.45-118.13)	105.84 (97.49-113.41)	101.08 (91.03-108.10)	**<0.001**
Plaque score	0.94 ± 1.02	0.11 ± 0.31	0.90 ± 1.01	2.06 ± 0.33	**<0.001**
Type of antidiabetic therapy					
No, %	35.4	60.2	33.4	7.1	**<0.001**
Insulin, %	4.5	3.8	4.9	4.9	0.811
OHA, %	34.4	19.9	40.1	42.9	**<0.001**
Insulin+OHA, %	26.2	16.5	22.2	45.7	**<0.001**
Hypercholesterolemia, %	11.5	11.9	10.6	12.5	0.798
Hypotriglyceridemia, %	24.6	23.3	25.2	25.0	0.861
Lipid-lowering therapy, %	28.2	28.8	26.1	31.0	0.488
DR, %	41.1	32.6	40.7	52.7	**<0.001**
DPN, %	29.4	14.8	29.8	47.3	**<0.001**

The use of bold emphasis is to mark the statistically significant indicators.

LCN-2, lipocalin-2; UACR, urinary albumin-to-creatinine ratio; BMI, body mass index; FPG, fasting plasma glucose; HbA1c, hemoglobin A1c; SBP, systolic blood pressure; DBP, diastolic blood pressure; TG, triglyceride; TC, total cholesterol; HDL-c, high-density lipoprotein cholesterol; LDL-c, low-density lipoprotein cholesterol; Sua, serum uric acid; Bun, blood urea nitrogen; Scr, serum creatinine; ARB, angiotensin receptor blocker; ACEI, angiotensin-converting enzyme inhibitor; OHA, oral hypoglycemic agent; DR, diabetic retinopathy; DPN, diabetic peripheral neuropathy.

In addition, a higher prevalence of diabetic retinopathy (DR) and diabetic peripheral neuropathy (DPN) was also observed in these T2DM patients with DN and/or CAP as compared with those without these complications, respectively (all *P* < 0.001). Similarly, a worse of renal function indexes including elevated UACR, BUN, and Scr was found in these T2DM individuals with DN and/or CAP compared with those without these complications (all *P* < 0.05). The average plaque score was much higher in the patients with DN and/or CAP in T2DM patients than in those without these complications (*P* < 0.001).

**Figure 1:**
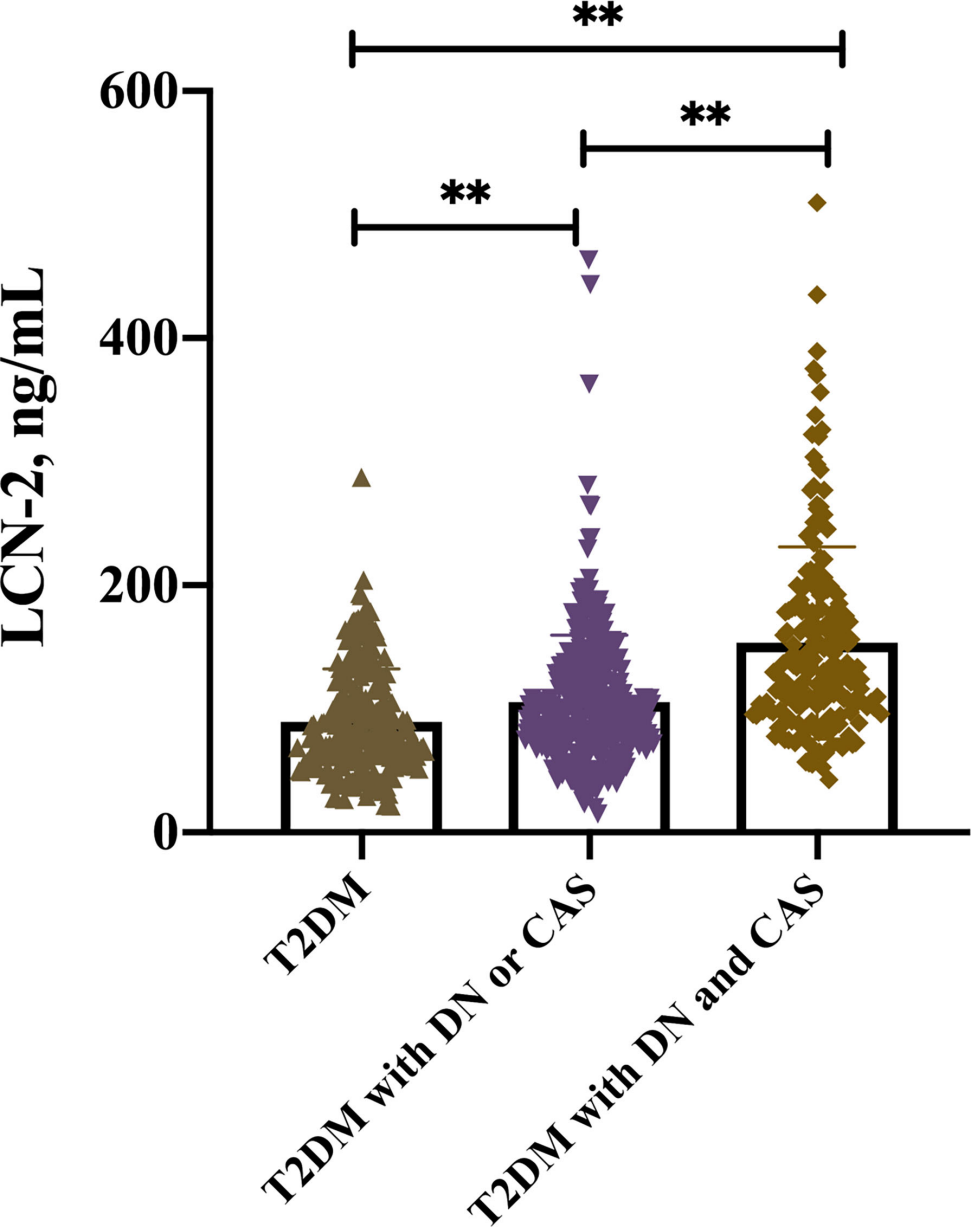
Serum LCN-2 levels in T2DM patients with or without relevant complications including DN and CAP, **P < 0.001.

Interestingly, multiple comparison results showed that serum LCN-2 levels were significantly increased in these T2DM patients with DN or CAP as compared with those without these complications [97.71 (71.49-130.13) *vs.* 77.29 (58.83-115.05) ng/ml, *P* < 0.05] (multiple comparison data not shown). Furthermore, higher serum LCN-2 levels were also observed in these T2DM patients with both DN and CAP as compared to those with one of these complications [131.37 (101.43-182.04) *vs.* 97.71 (71.49-130.13) ng/ml, *P* < 0.05] (**Table 2** and **Figure 1**) (multiple comparison data not shown), suggesting that LCN-2 may be related to the progress of disease in these patients with T2DM.

### Serum Lipocalin-2 Levels Are Closely Correlated With a Cluster of Renal Function Parameters and Carotid Atherosclerotic Plaque in Patients With Type 2 Diabetes Mellitus

We next investigated the relationship between serum LCN-2 levels and a cluster of anthropometric parameters and renal function parameters. Correlation analysis showed a significant positive association of serum LCN-2 levels with diabetes duration, HbA1c, SBP, hypertension, CAP, DN, and relevant renal function parameters including Sua, Scr, and UACR, respectively (all *P* < 0.05), and serum LCN-2 was negatively correlated with HDL-c (*P* < 0.001), after the adjustment for age. Furthermore, the positive correlation of serum LCN-2 with these parameters, except for HDL-c, Sua, and DN, remained significant even after the adjustment for age and diabetes duration (**Table 3**).

**Table 3:** Partial correlation of serum LCN-2 with other risk factors of DN and CAP.

	Serum LCN-2		Serum LCN-2 (age-adjusted)		Serum LCN-2 (age- and T2DM duration-adjusted)	
Variables	*r*	*P*	*r*	*P*	*r*	*P*
Age	0.198	**<0.001**				
T2DM duration	0.188	**<0.001**	0.133	**<0.001**		
BMI	-0.031	0.404	-0.019	0.598	-0.013	0.720
HbA1c^*^	0.047	0.201	0.108	**0.003**	0.106	**<0.001**
SBP	0.168	**<0.001**	0.115	**0.002**	0.099	**0.004**
DBP	0.016	0.672	0.022	0.545	0.027	**0.007**
TG^*^	0.029	0.428	0.064	0.081	0.066	0.458
TC^*^	-0.041	0.260	-0.015	0.680	-0.016	0.072
HDL-c^*^	-0.096	**0.009**	-0.140	**<0.001**	-0.147	0.667
LDL-c^*^	-0.076	**0.039**	-0.055	0.134	-0.052	0.161
Sua^*^	0.056	0.124	0.074	**0.044**	0.069	0.060
Bun^*^	0.099	**0.007**	0.041	0.262	0.037	0.314
Scr^*^	0.187	**<0.001**	0.170	**<0.001**	0.161	**<0.001**
UACR^*^	0.260	**<0.001**	0.235	**<0.001**	0.201	**<0.001**
Hypertension	0.166	**<0.001**	0.099	**0.007**	0.091	**0.013**
Plaque score	0.399	**<0.001**	0.354	**<0.001**	0.347	**<0.001**
CAP	0.412	**<0.001**	0.370	**<0.001**	0.361	**<0.001**
DN	0.165	**<0.001**	0.140	**<0.001**	0.068	0.064

^*^Log transformed before analysis. The use of bold emphasis is to mark the statistically significant indicators.

To clarify whether DN is correlated with LCN-2 independently adjusted by CAP, we have further analyzed the relationship of serum LCN-2 with DN, UACR, and renal function parameters by partial correlation analysis. As shown in **Supplementary Table 1**, UACR was positively correlated with LCN-2 levels when adjusted by CAP, suggesting that serum lipocalin-2 levels are independently associated with early-stage renal damage.

### Serum Lipocalin-2 Levels Are Independently Associated With Early-Stage Renal Damage and CAP in Patients With T2DM

To further determine whether serum LCN-2 was independently associated with renal damage and CAP in patients with T2DM, the multinomial logistic regression analysis was performed. Results from the multinomial logistic regression analysis between the T2DM group and the DN or CAP group indicated that serum LCN-2 was found to be independently associated with DN or CAP [*OR* 2.873 (*95%CI* 1.798-4.590), *P* < 0.001], together with age and the T2DM duration after the adjustment for basic factors (Model 1). Furthermore, serum LCN-2 was found to be independently associated with DN or CAP, together with hypertension and DPN after the adjustment for complications and past history (Model 2). In addition, serum LCN-2 was found to be independently associated with DN or CAP, together with ACEI/ARB and antidiabetic therapy, except lipid-lowering therapy after the adjustment for hypoglycemic and lipid-lowering variables (Model 3). Finally, to adequately determine whether serum LCN-2 was independently associated with DN and/or CAP in patients with T2DM, a full model was developed and adjusted by variables with *P* < 0.05 in models 1-3 (Model 1-3) and all the parameters with a significant correlation with serum LCN-2. Interestingly, the association between serum LCN-2 and DN or CAP still remained significant (full model, **Table 4**).

**Table 4:** Multinomial logistic regression showing whether LCN-2 was an independent influencing factor for DN and CAP.

	T2DM group *vs.* DN or CAP group		T2DM group *vs.* DN with CAP group	
Variables	*OR* (95%CI)	*P*	*OR* (95%CI)	*P*
**Model 1**				
Age	1.050 (1.028-1.072)	**<0.001**	1.089 (1.054-1.125)	**<0.001**
Gender	1.013 (0.951-1.078)	0.464	1.360 (0.615-3.009)	0.448
T2DM duration	1.080 (1.055-1.106)	**<0.001**	1.093 (1.068-1.119)	**<0.001**
BMI	1.005 (0.993-1.017)	0.696	1.001 (0.913-1.097)	0.985
SBP	1.223 (0.713-2.098)	0.414	1.016 (1.000-1.033)	**0.045**
Smoking	0.689 (0.426-1.114)	0.129	0.420 (0.209-0.847)	**0.015**
LCN-2^*^	2.873 (1.798-4.590)	**<0.001**	18.115 (9.259-35.441)	**<0.001**
**Model 2**				
Hypertension	0.606 (0.422-0.869)	**0.007**	0.264 (0.167-0.419)	**<0.001**
DR	0.861 (0.595-1.246)	0.427	0.708 (0.442-1.133)	0.150
DPN	0.414 (0.265-0.648)	**<0.001**	0.182 (0.107-0.310)	**<0.001**
LCN-2^*^	1.996 (1.382-2.884)	**<0.001**	12.012 (7.124-20.253)	**<0.001**
**Model 3**				
ACEI/ARB	0.595(0.412-0.860)	**0.006**	0.274 (0.167-0.495)	**<0.001**
Antidiabetic therapy				
Insulin	0.428 (0.179-1.026)	0.057	0.138 (0.038-0.495)	**0.002**
OHA	0.264 (0.171-0.407)	**<0.001**	0.050 (0.024-0.103)	**<0.001**
Insulin+OHA	0.390 (0.242-0.628)	**<0.001**	0.040 (0.019-0.084)	**<0.001**
Lipid-lowering therapy	1.211 (0.813-1.802)	0.346	0.992 (0.588-1.674)	0.977
LCN-2^*^	2.346 (1.598-3.444)	**<0.001**	15.268 (8.711-26.761)	**<0.001**
**Full model**				
Age	1.058 (1.034-1.084)	**<0.001**	1.105 (1.065-1.147)	**<0.001**
T2DM duration	1.078 (1.049-1.108)	**<0.001**	1.087 (1.058-1.118)	**<0.001**
Smoking	0.598 (0.374-0.957)	**0.032**	0.312 (0.159-0.611)	**0.001**
Hypertension	0.998 (0.604-1.648)	0.993	0.686 (0.344-1.365)	0.283
DPN	0.857 (0.466-1.574)	0.619	0.700 (0.306-1.601)	0.397
ACEI/ARB	0.796 (0.484-1.311)	0.371	0.732 (0.343-1.560)	0.419
Insulin	0.736 (0.247-2.199)	0.584	0.347 (0.071-1.694)	0.191
OHA	0.842 (0.485-1.462)	0.542	0.404 (0.164-0.991)	**0.048**
Insulin+OHA	1.125 (0.606-2.089)	0.709	0.530 (0.198-1.420)	0.207
HbA1c^*^	0.531 (0.202-1.398)	0.200	1.303 (0.314-5.404)	0.716
Scr^*^	0.724 (0.197-2.653)	0.625	0.456 (0.079-2.637)	0.381
UACR^*^	1.770 (1.392-2.250)	**<0.001**	2.699 (2.033-3.583)	**<0.001**
LCN-2^*^	3.128 (1.903-5.142)	**<0.001**	18.354 (8.843-38.093)	**<0.001**

^*^Log transformed before analysis. The use of bold emphasis is to mark the statistically significant indicators.

Model 1, adjusted by basic factors including age, gender, T2DM duration, BMI, SBP, and smoking;

Model 2, adjusted by complication and past history including hypertension, DR, and DPN;

Model 3, adjusted by hypoglycemic and lipid-lowering variables;

Full model, adjusted by variables with P < 0.05 in models 1–3 and all the parameters with significant correlation with serum LCN-2.

Furthermore, to clarify the relationship between serum LCN-2 and early-stage renal damage in T2DM patients with DN and/or CAP, a pairwise comparison has been performed between 4 groups of these subjects. As shown in **Supplementary Table 2**, **3** serum LCN-2 levels were significantly increased in T2DM patients with both DN and CAP as compared with those without these complications, as well as those with CAP but without DN (*P* < 0.001). Furthermore, a higher serum LCN-2 level was observed in T2DM patients with CAP as compared to those with DN. By contrast, a lower UCAR value was observed in T2DM patients with CAP than those with DN. These data suggested that the elevated LCN-2 levels are independently associated with the early-stage renal damage in patients with T2DM when adjusted by CAP. Furthermore, DN, UACR, and Scr were all in correlation with LCN-2 levels when adjusted by CAP in the correlation analysis (*P* < 0.001) (**Supplementary Table 1**).

Likewise, the data from multinomial logistic regression analysis between the T2DM group and DN plus the CAP group also demonstrated that serum LCN-2 was independently associated with DN and CAP in four different models (**Table 4**). Taken together, these data indicated that serum LCN-2 is independently associated with DN and CAP in patients with T2DM.

## Discussion

Clinical and epidemiological studies demonstrated that dyslipidemia, smoking, diabetes mellitus, hypertension, obesity, psychosocial stress, poor diet, and physical inactivity were conventional risk factors related to the incidence of CVD, while diabetes has long been considered as an independent contributing factor for the pathogenesis of CVD. In the current study, we investigated the relationship of LCN-2 with early-stage renal damage and CAP in patients with T2DM. In this study, we provided the first clinical evidence showing that T2DM patients with DN had a higher incidence of CAP than those without DN. In addition, serum LCN-2 levels are significantly increased and associated with early-stage renal damage and CAP in patients with T2DM. These findings indicated that early-stage renal damage is a risk factor associated with the incidence of CAP in patients with T2DM, and serum LCN-2 may be a biomarker that is related to the early-stage renal damage and pathogenesis of CAP in patients with T2DM.

Epidemiological studies indicated that T2DM is becoming an epidemic disease, and more than 11.2% of the total population were diagnosed with diabetes in China (20). More frighteningly, except for its heavy burden on public health, T2DM is considered as one of the most important risk factors related to CVD, which increases all-cause mortality (21). DN, one of general diabetic microvascular complications, is the main reason leading to chronic kidney disease. Regional cross-sectional studies reported that the DN prevalence ranged from 29.6% to 49.6% in mainland China (22–24), and the overall prevalence of DKD ranged from 17% to 35% in the United States from 1998 and 2014, as evaluated by the National Health and Nutrition Examination Survey (NHANES) (25). Compared with the data from the United States and Hong Kong (26, 27), the albuminuria rates in mainland China obviously increased. Therefore, we speculated that DN presents an important role to promote and increase the morbidity of CVD. This is supported by the fact that there is gradual increase of morbidity and mortality in CVD in the Chinese population (28). In the present study, the prevalence of DN in patients with T2DM was 50.87%, and a higher incidence of CAP was observed in T2DM patients with DN compared with those without DN, suggesting that DN may be one of risk factors that accelerated the development of CVD in patients with T2DM.

Previous studies indicated that both DN and CAP are common vascular complications of diabetes, and both of these two vascular complications are reciprocal causation to promote the development of disease (29). DN is independently associated with carotid plaque formation, and renal insufficiency could accelerate the occurrence of AS (30). Therefore, looking for a suitable biomarker related to DN and CAP in patients with T2DM is very important for diagnosing and monitoring the development of disease. LCN-2, also called NGAL, is markedly induced by multiple stresses (8, 31). Previous studies showed that LCN-2 levels are markedly elevated in patients with CVD, including AS and heart failure, but not in healthy controls (9, 32). Consistent with previous studies, our data also indicated that serum LCN-2 levels were significantly increased and associated with the incidence of CAP in patients with T2DM. Interestingly, serum LCN-2 levels were markedly increased in T2DM patients with DN as compared to those without relevant complication and independently associated with renal function factors including Scr and UACR, a conventional parameter of early-stage renal damage. In addition, serum LCN-2 levels were gradually increased from T2DM patients without complication to those with DN and/or CAP. Taken together, these data demonstrated that LCN-2 may be related to the progress of disease from DN to CAP in patients with T2DM.

In conclusion, the present study is the first to present the relationship between circulating LCN-2 levels and DN and CAP in patients with T2DM. Our data demonstrated that early-stage renal damage is a risk factor associated with the incidence of CAP in patients with T2DM. Furthermore, serum LCN-2 is significantly increased and associated with early-stage renal damage as well as the incidence of CAP in patients with T2DM, and LCN-2 may be a potential biomarker related to the progression of disease in patients with T2DM.

## Data Availability Statement

The raw data supporting the conclusions of this article will be made available by the authors, without undue reservation.

## Ethics Statement

The studies involving human participants were reviewed and approved by Human Research Committee Affiliated Hospital of the Third Affiliated Hospital of Wenzhou Medical University (Ruian People’s Hospital) approved the study. The patients/participants provided their written informed consent to participate in this study.

## Author Contributions

JG drafted the manuscript. QY, YiZ, and WX have participated in data collection. JG, YS, and NY performed the statistical analyses. WX, YiZ, NY, and YY have performed the laboratory measurements. YuZ, ZL, and HY have edited and revised the manuscript. All authors have read and approved the final manuscript submitted.

## Funding

This research was supported by China National Funds for Distinguished Young Scientist (No: 81925004 to ZL), National Natural Science Foundation of China (General program no: 81870317 to ZL).

## Conflict of Interest

The authors declare that the research was conducted in the absence of any commercial or financial relationships that could be construed as a potential conflict of interest.

## Publisher’s Note

All claims expressed in this article are solely those of the authors and do not necessarily represent those of their affiliated organizations, or those of the publisher, the editors and the reviewers. Any product that may be evaluated in this article, or claim that may be made by its manufacturer, is not guaranteed or endorsed by the publisher.
