# Supplementary material for: Serum Lipocalin-2 Levels Are Increased and Independently Associated With Early-Stage Renal Damage and Carotid Atherosclerotic Plaque in Patients With T2DM
Source: Front Endocrinol (Lausanne). 2022 Apr 25;13:855616. doi: 10.3389/fendo.2022.855616 (PMC9081837; doi:10.3389/fendo.2022.855616)
Supplement: Supplementary file 1 [file Table_1.docx]

**Supplementary Table 1** Partial correlation of serum LCN-2 with DN or UACR adjusted by CAP

|  | Serum LCN-2 (CAP-adjusted) | |
| --- | --- | --- |
| Variables | *r* | *P* |
| DN | 0.125 | 0.001 |
| UACR^*^ | 0.179 | < 0.001 |
| Sua^*^ | 0.055 | 0.131 |
| Bun^*^ | 0.041 | 0.260 |
| Scr^*^ | 0.148 | < 0.001 |

Note. ^*^ Log transformed before analysis.

**Supplementary Table 2** Comparison between groups with DN without CAP and the others

| Variables | T2DM group  （n=236） | DN without CAP group  （n=197） | DN with CAP group  （n=184） | CAP without DN  （n=132） | *P* |
| --- | --- | --- | --- | --- | --- |
| LCN-2, ng/mL | 77.29 (58.83-115.05) | 85.87 (63.80-113.89) | 131.37 (101.43-182.04) | 108.97(82.31-144.13) | < 0.001 |
| UACR, mg/g | 11.35 (6.98-17.76) | 35.92 (14.04-86.78) | 68.90 (32.21-277.38) | 12.45(7.54-19.08) | < 0.001 |

**Supplementary Table 3** Pairwise comparison between groups of LCN-2

| Pairwise comparison | *SE* | *P* |
| --- | --- | --- |
| T2DM *vs.* DN without CAP | 20.880 | 0.221 |
| T2DM *vs.* CAP without DN | 23.516 | < 0.001 |
| T2DM *vs.* DN with CAP | 21.278 | < 0.001 |
| DN without CAP *vs.* CAP without DN | 24.337 | < 0.001 |
| DN without CAP *vs.* DN with CAP | 22.182 | < 0.001 |
| CAP without DN *vs.* DN with CAP | 24.679 | 0.001 |
